# APNG as a prognostic marker in patients with glioblastoma

**DOI:** 10.1371/journal.pone.0178693

**Published:** 2017-06-29

**Authors:** Sigurd Fosmark, Sofie Hellwege, Rikke H. Dahlrot, Kristian L. Jensen, Helene Derand, Jesper Lohse, Mia D. Sørensen, Steinbjørn Hansen, Bjarne W. Kristensen

**Affiliations:** 1 Department of Pathology, Odense University Hospital, Odense, Denmark; 2 Department of Clinical Research, University of Southern Denmark, Odense, Denmark; 3 Department of Oncology, Odense University Hospital, Odense, Denmark; 4 DAKO Denmark A/S, Glostrup, Denmark; University of Alabama at Birmingham, UNITED STATES

## Abstract

**Aim:**

Expression of the base excision repair enzyme alkylpurine-DNA-N-glycosylase (APNG) has been correlated to temozolomide resistance. Our aim was to evaluate the prognostic value of APNG in a population-based cohort with 242 gliomas including 185 glioblastomas (GBMs). Cellular heterogeneity of GBMs was taken into account by excluding APNG expression in non-tumor cells from the analysis.

**Methods:**

APNG expression was evaluated using automated image analysis and a novel quantitative immunohistochemical (IHC) assay (qIHC), where APNG protein expression was evaluated through countable dots. Non-tumor cells were excluded using an IHC/qIHC double-staining. For verification, APNG was measured by a quantitative double-immunofluorescence (IF) assay. As validation APNG mRNA expression was evaluated using independent TCGA data.

**Results:**

Using qIHC, high levels of APNG were associated with better overall survival (OS) in univariate (HR = 0.50; *P* < 0.001) and multivariate analysis (HR = 0.53; *P* = 0.001). Patients with methylated MGMT promoters and high APNG expression demonstrated better OS, than patients with methylated MGMT promoters and low APNG expression (HR = 0.59; *P* = 0.08). Retesting the cohort using IF showed similar results in both univariate (HR = 0.61; *P* = 0.002) and multivariate analysis (HR = 0.81; *P* = 0.2). The results were supported by data from the TCGA database.

**Conclusions:**

Using two different assays combined with quantitative image analysis excluding non-tumour cells, APNG was an independent prognostic factor among patients with a methylated MGMT promoter. We expect that APNG qIHC can potentially identify GBM patients who will not benefit from treatment with temozolomide.

## Introduction

Glioblastoma multiforme (GBM) is the most common and malignant primary brain tumor in adults. The prognosis has improved from a median survival of 12.1 months with surgery and radiotherapy alone to 14.6 months with today’s standard treatment, which includes concomitant and adjuvant temozolomide (TMZ) [1–3]. Additional groups [4–8] have reported that patients with a methylated MGMT promoter benefit more from treatment with TMZ than patients with an un-methylated promoter, especially in the elderly GBM patients [9, 10]. However, some patients with methylated MGMT status do not respond to TMZ therapy, indicating that MGMT status alone is not responsible for resistance towards TMZ in patients with GBMs. Other mechanisms have been investigated, and studies suggest that alkylpurine-DNA-N-glycosylase (APNG), also known as DNA methylpurine-N-glycosylase (MPG), can sensitize several types of cancer cells towards TMZ [11, 12].

APNG, a DNA repair enzyme, is part of the base excision repair (BER) system, which repairs the N3-methyladenine and N7-methylguanine adducts created by alkylating chemotherapeutic agents [13–15]. These methylations are the most abundant cytotoxic lesions produced by chemotherapeutics such as TMZ and comprise approximately 80% of the adducts [15–17]. Previously, APNG has shown promising potential as a predictive [3] and prognostic [18] marker in patients with GBMs. Agnihotri et al. used several in vitro assays as well as a GBM xenograft model and showed that loss of APNG sensitizes cells to TMZ. Subsequently, the authors performed an immunohistochemical (IHC) analysis of APNG in 244 GBM specimens by including several sample sets [3]. For each cohort, patients were dichotomized into a positive and a negative population, and univariate analyses were performed. In a large group of patients treated with radiotherapy alone, APNG was not associated with overall survival (OS). However; in the EORTC-NCIC cohort a median OS of 12 months was found for APNG-positive patients compared to 16 months for APNG-negative patients [3]. Liu et al analyzed the expression of APNG in 128 glioma patients using IHC and showed that the survival rate was significantly shorter for glioma patients with positive APNG than for those with a negative APNG status [18]. Based on these studies, APNG appeared to be an important biomarker in GBM patients treated with TMZ.

The aim of this study was to investigate the association between OS and APNG status in a retrospective well annotated patient cohort. Further, the level of APNG was examined using both a quantitative immunofluorescence (IF) method used previously in our lab [19, 20], and the new method of dot technique in quantitative immunohistochemistry (qIHC) [21]. qIHC is a novel methodology developed by DAKO, which combined with image analysis, allows for quantitation of the expression of proteins in paraffin embedded tissue samples using bright field assessments [22]. The current IF method has previously been used successfully in other biomarker studies on the same cohort [19, 20]. In the present study both qIHC and the IF techniques were further developed in order to identify both tumor- and non-tumor cells. Exclusion of non-tumor cells removed any non-tumor APNG contribution, and hence the cellular heterogeneity of GBMs was taken into account and excluded from the analysis.

## Methods

### Ethics

The official Danish ethical review board named the Regional Scientific Ethical Committee of the Region of Southern Demark approved the use of human glioma tissue (permission J. No. S-2011 0022) in the current study.

### Patient material

Patients from the Region of Southern Denmark, who were diagnosed with a primary glioma between 1 January 2005 and 31 December 2009, were included in the population-based cohort. The material used is well-described and investigated in several studies [19, 20, 23–25].

A total of 242 patients had sufficient viable tissue for further analysis. In the qIHC assay five sections were subsequently excluded due to small amounts of tumor tissue. Of the samples included, 3 were WHO grade I, 23 WHO grade II, 25 WHO grade III and 186 WHO grade IV. In the IF assay 19 tumor samples were excluded due to technical challenges, mainly unspecific staining. The remaining 223 tumor samples were as follows: 3 WHO grade I, 21 WHO grade II, 21 WHO grade III, and 178 WHO grade IV tumors. Tumor samples were classified by two neuropathologists in accordance with the WHO classification of tumors of the central nervous system [26]. Patient characteristics are described in Table 1.

**Table 1:** Patient characteristics.

	WHO Grade II				WHO Grade III				WHO Grade IV			
	qIHC		IF		qIHC		IF		qIHC		IF	
	n	(%)	n	(%)	n	(%)	n	(%)	n	(%)	n	(%)
**Subjects**	23	(10)	21	(10)	25	(11)	21	(10)	186	(79)	178	(80)
**Age** (median)	46		46		58		58		65		65	
**Gender**												
Male	13	(57)	12	(57)	18	(72)	15	(71)	108	(58)	102	(57)
Female	10	(43)	9	(43)	7	(28)	6	(29)	78	(42)	6	(43)
**PS**												
0–1	18	(78)	15	(71)	19	(76)	16	(76)	117	(63)	110	(62)
2–4	5	(22)	6	(29)	6	(24)	5	(24)	69	(37)	68	(38)
**APNG**												
dots/cell	0.32				0.42				0.31			
AF			0.50				0.67				0.69	
**Dead**	14	(61)	12	(57)	23	(92)	19	(90)	177	(95)	170	(95)
**OS** (months)	58		58		14		21		10		10	

Abbreviations: PS ECOG performance status; AF area fraction; qIHC quantitative immunohistochemistry; IF immunofluorescence

We used data from two different cohorts generated by the TCGA Research Network (http://cancergenome.nih.gov/) for supplementary analysis on mRNA APNG. Information regarding APNG, based on the level of mRNA, age, gender, IDH1 and MGMT status were obtained. Data in the first cohort was derived from RNA sequencing analyses [27], and a total of 620 patients were identified; of these 226 were WHO grade II gliomas, 244 were WHO grade III and 150 were GBMs. This cohort was used for comparison of the APNG mRNA level between the different WHO grades only. Data in the other cohort was generated using Affymetrix U133A [28], and a total of 497 patients with WHO grade IV tumors were identified. This cohort was used for multivariate analysis. Both cohorts have been described previously [27, 28], brief patient characteristics are shown in S1 and S2 Tables.

### Immunohistochemistry

For the qIHC assay, sections from FFPE tissues were stained using qIHC and the Dako EnVisionTM+ system (Dako, Glostrup, Denmark). All stainings were performed at Dako Denmark. Slides were de-paraffinized via baths of 2x5 min xylene, 2x2 min 96% ethanol, 2x2 min 70% ethanol and finally transferred to demineralized water. Target retrieval was performed in a buffer of 5 mM Na-Hepes, pH 8.0, at 97°C for 20 min, in a Dako PT-link module. To block endogenous peroxidase the sections were treated with Dako blocking reagent with additive. Endogenous peroxidase block was performed initially (10 min) and after qIHC precipitation reaction (20 min). Automated staining was performed on an Autostainer Link 48 (Dako). Monoclonal rabbit anti-APNG (Abcam clone ab55461) (1:5000) was used as primary antibody for qIHC (20 min). A cocktail of CD31 (M0823 (Dako), Clone JC70A) (1:30), CD45 (M0701 (Dako), Clones 2B11 + PD7/26) (1:75) and Iba1 (Wako Cat. #019–19741) (1:8000) antibodies, which identify endothelial cells, leukocytes, and microglia was used as primary antibodies for standard IHC/3'3-diaminobenzidine tetra-hydrochloride (DAB) EnVision Flex staining (20 min). qIHC staining was performed as described previously [22]. Conceptually, a polyclonal goat anti-rabbit antibody conjugated to horseradish peroxidase (HRP) was used as secondary antibody. Polyclonal rabbit anti-fluorescein isothiocyanate (FITC) conjugated to calf intestine alkaline phosphatase (CIP) was used as tertiary conjugate to produce colored liquid permanent red (LPR) dots for bright field microscopy. IHC staining with Dako Envision Flex+ Mouse linker system was performed according to manufacturer’s instructions. In short, sections were incubated with Dako EnVisionTM FLEX+ mouse linker for 15 minutes followed by incubation with Dako EnVisionTM FLEX/HRP for an additional 20 minutes. For visualization of staining, the sections were treated with DAB. Finally, slides were counterstained with haematoxylin, rinsed with water, dehydrated in 99.9% ethanol and cover slipped with Sakura Tissue-Tek Film Coverslipper.

Double fluorescence immunohistochemistry was performed on the Dako Autostainer plus platform (DAKO Denmark A/S, Glostrup Denmark) in the Department of Pathology, Odense University Hospital. Sections were dewaxed with xylene and rehydrated with ethanol. Heat epitope retrieval (HIER) was performed using a microwave oven in TEG-buffer for 15 minutes at 100°C. Endogen peroxidase activity was blocked/reduced with 5-minute incubation in hydrogen peroxidase. The sections were incubated with the primary antibody, monoclonal mouse anti-APNG antibody (Abcam clone ab55461), at a dilution of 1:12000. Detection was performed using DAKO CSA II, Biotin-Free Catalyzed Amplification System. A second HIER was performed, as well as a second blocking of endogen peroxidase with hydrogen peroxide. The sections were then incubated with a triple-cocktail consisting of antibodies against CD31 (M0823 (Dako), Clone JC70A) (1:50), CD45 (M0701 (Dako), Clones 2B11 + PD7/26) (1:1600) and Iba1 (Wako Cat. #019–19741) (1:12000). Detection was performed using Perkin Elmer TSATM, Plus Cyanine 5 System. Nuclear counterstaining was performed using VECTASHIELD Mounting Medium containing DAPI.

Regarding both qIHC and IF, the stainings were compared with conventional chromogenic IHC stainings to ensure specific reaction patterns.

### Image analysis

For qIHC all slides were scanned using an Aperio Scanscope CS system (Aperio Technologies Inc., Vista, CA, USA), at 20x magnification level. All images were analyzed using the Aperio Genie pattern recognition. The system was trained to identify 3 different categories based on morphology: Empty glass, tumor and normal tissue, enabling the Genie algorithm to distinguish between the three. The Genie Algorithm was trained by an experienced assessor using a test-set consisting of 20 gliomas. To increase the precision of the analyzing tool, the test-set was trained for 25,000 iterations. Tumor tissue identified by the Genie pattern recognition tool was analyzed using the Indicalab ISH algorithm v. 1.2 (IndicaLab, Corrales, NM, USA). The sensitivity of the algorithm was fine-tuned by comparing qIHC slides with conventional IHC slides. All slides were evaluated in the Aperio ImageScope software (Aperio Technologies Inc., Vista, CA, USA) by a trained observer and reviewed by a certified neuropathologist prior to analysis. This enabled visual assessment of each individual slide, as well as exclusion of predefined areas from analysis based on the following exclusion criteria: 1) low cellularity, 2) necrosis, 3) larger vessels 4) artifacts. Remaining areas were labeled as regions of interest (ROI) and included in the analysis. Classification of areas within ROIs was based on recognition of the nuclear haematoxylin stain and exclusion of non-tumor cells exhibiting DAB positivity. APNG protein expression was visualized as individual red chromogenic dots, each representing a predefined amount of protein determined during assay development. Only APNG protein expression in nuclei was included. This was determined by the distance of dots to nuclei and was set to include only dots within or with minimal distance to nuclei borders. The number of dots per cell and a mean dots/cell was calculated for each slide.

For IF all images were obtained using a Leica DM6000B epifluorescense microscope, with an Olympus DM72 1.4 Mega Pixel (1024 x 1360) high sensitivity CCD camera. Images were classified based on the RGB three color model, and for each pixel the intensity threshold of three different colors, red, green, and blue, was defined in the Visiomorph^™^ software module (Visiopharm, Hørsholm, Denmark). Then uniform random sampling (meander fraction based) was performed at 20x magnification as follows. Initially a bright-field image was taken of the stained tumor sections at 1.25x magnification. Subsequently the ROI’s were manually outlined and the sampling performed. The exclusion criteria were the same as for qIHC; additionally the images had to be well-exposed with limited unspecific background staining in all of the three color bands. It was pre-defined that a minimum of 6 images should be available for each tumor, or the tumor would be excluded from the analysis. An initial sampling fraction between 5% and 30% was therefore used depending on area of tumor tissue. Exposure time for DAPI and Cy5 were the same for all six staining-runs. Exposure time for FITC was calibrated to avoid staining-run variation for APNG as previously described [3]. Areas with non-tumor cells were excluded using the triple-cocktail leaving only tumor cells for further evaluation. APNG positive nuclear areas were then defined as areas with intensity higher than the defined threshold identifying positive staining, and based on the DAPI staining APNG positive and negative nuclear areas were identified. Outcome variables were area fraction of APNG-positive tumor cell nuclei (AF) and mean intensity of APNG in tumor cell nuclei (MI). A certified neuropathologist was responsible for the fluorescence exposure and the training of the image analysis software. The training was performed on multiple images.

### Statistical analysis

Data was described using frequency tables. Correlation between IDH1 status, APNG and survival was investigated using t-test. Median APNG protein expression was used as pre-defined cutoff values. Patients with WHO grade I tumors were excluded from analyses due to the limited number of patients, thus 234 (qIHC) and 220 (IF) patients were included in the final analyses. Overall survival, being defined as the time from primary surgery until death or censoring (November 2014) was represented by Kaplan-Meyer curves. The survival functions were analyzed for differences by the log-rank test. Cox proportional hazard regression analyses were performed for patients with GBM. Only clinical variables which previously have been reported to be prognostic in this cohort were included [23]. Optimal cutoff analyses were carried out as described in [20], and values confirmed using ROC curves. All analyses were performed in STATA version 11 (StataCorp LP, College Station, Texas, USA) computer software.

## Results

### Staining patterns in qIHC

**Fig 1:**
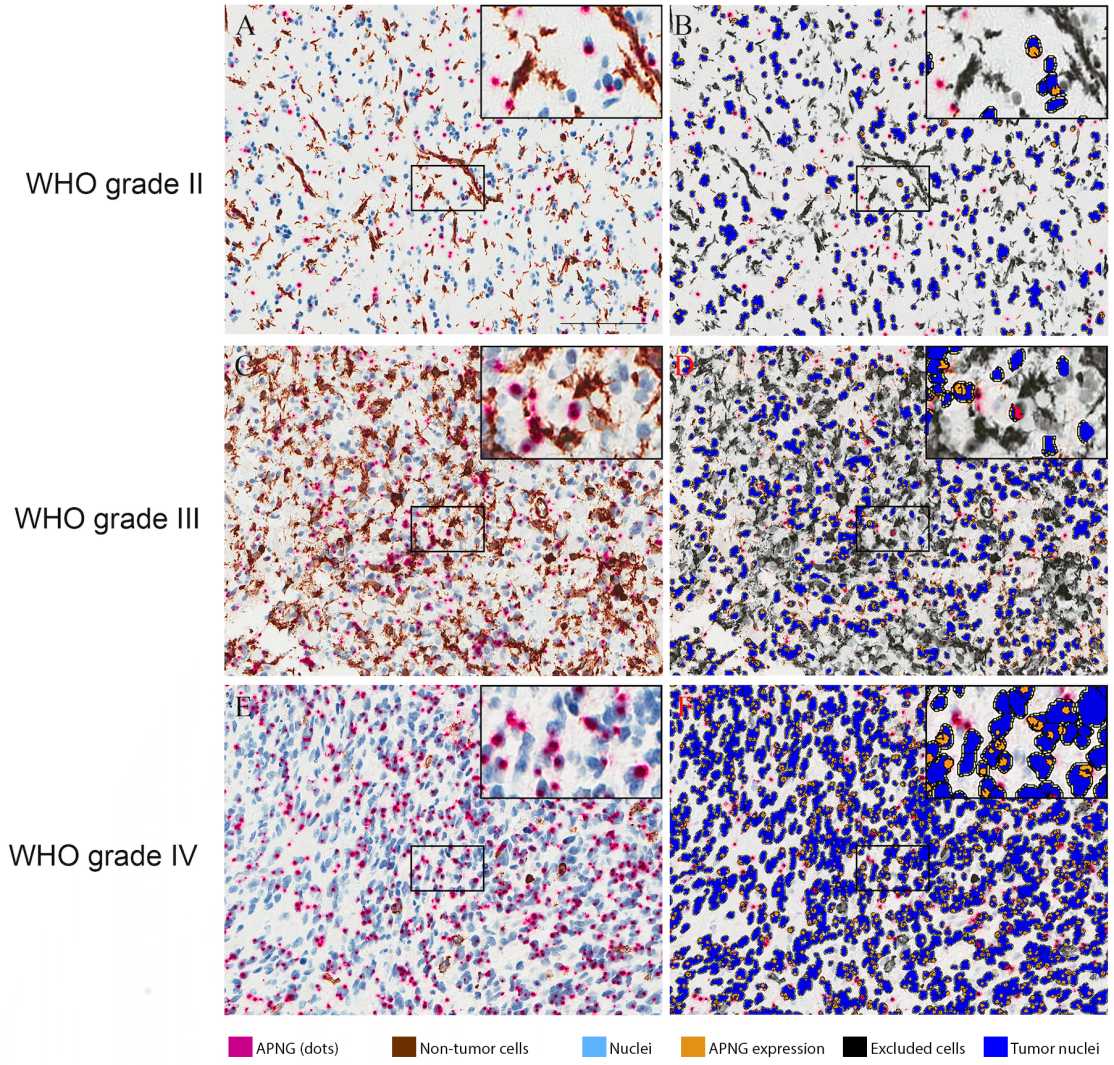
Images of APNG staining in different tumor subtypes. APNG expression visualized using quantitative immunohistochemistry (qIHC). Endothelial cells, leukocytes and microglia/macrophages were excluded using co-stainings. A, C, E: APNG expression in the nuclei is visualized and quantified by the number of red dots. Non-tumor cells were stained with DAB. IHC stainings were evaluated by 1) exclusion of non-tumor cells (DAB) and 2) visualization of APNG expression (red dots) in the tumor cells. B, D, F: Non-tumor cells were automatically excluded and APNG analysis was applied on tumor cells; nuclei are blue and APNG expression visualized as qIHC dots are orange. Scalebar: 100 μm.

Chromogenic stains showed APNG staining as clear, red dots in both tumor and non-tumor cells, and it was seen in all WHO grades, mainly in or in close proximity to nuclei. The few dots observed outside of cells were automatically excluded in accordance with the analysis algorithm. Exclusion markers in the triple cocktail containing CD31, CD45 and Iba-1 were seen as conventional chromogenic brown staining in the cytoplasm and in the membrane. The excluded non-tumor cells were frequently located around vessels and areas with necrosis or bleeding (Fig 1).

### Staining patterns in IF

**Fig 2:**
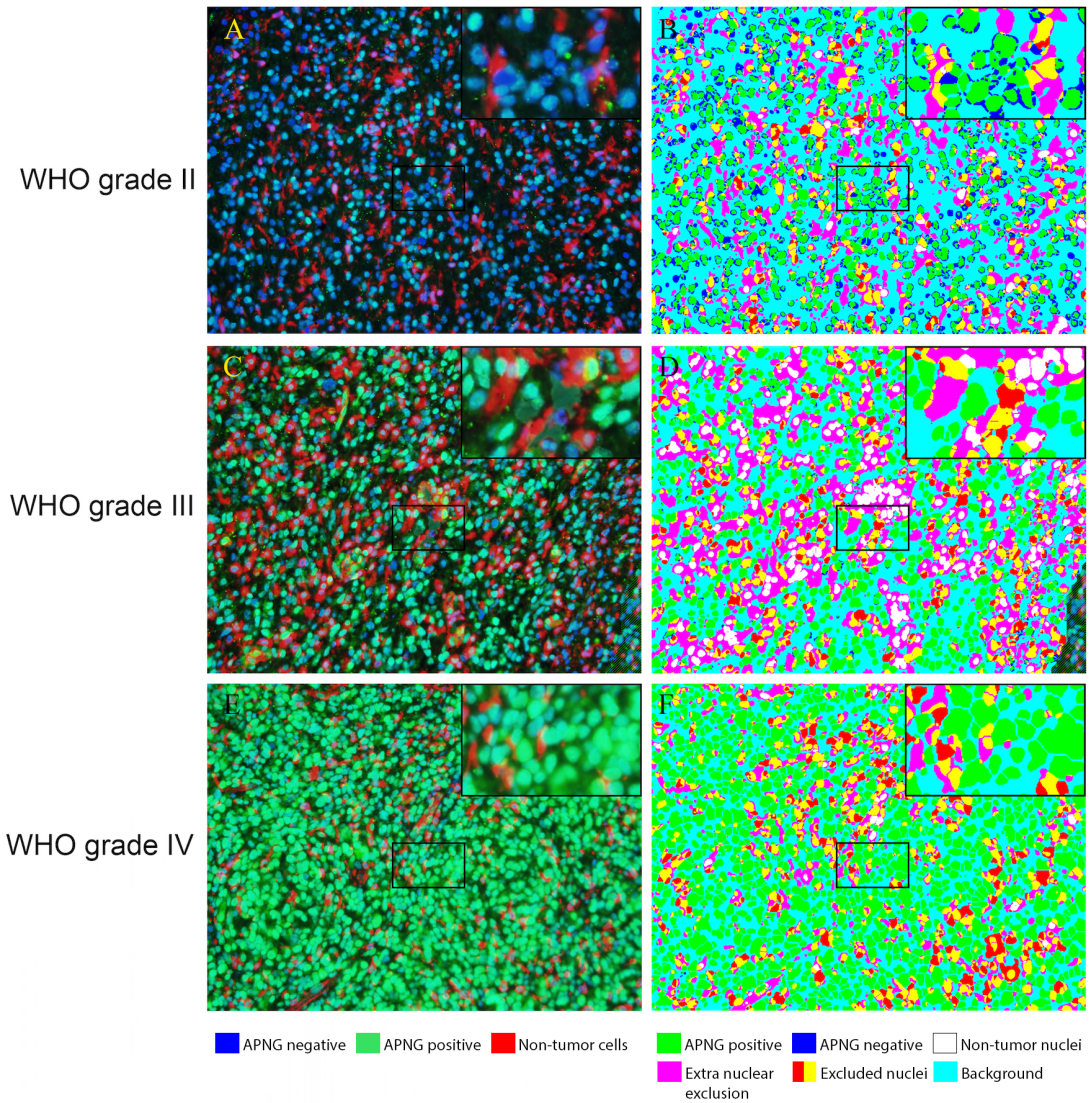
Staining of APNG and the corresponding processed images. APNG expression visualized using quantitative immunofluorescence (IF). Endothelial cells, leukocytes and microglia/macrophages were excluded using co-stainings. APNG negative (blue) and positive (green) nuclei were identified. Non-tumor cells are red. In B, D and F the image analysis software identifies the cells as follows: APNG-positive tumor cell nuclei (green), APNG-negative tumor nuclei (blue), non-tumor cell nuclei (white), extra-nuclear exclusion cocktail staining (pink), excluded nuclei (red and yellow) and background (turquoise).

Positive APNG-staining, represented as a green fluorescence staining, was observed in the nuclei in all WHO grades. Nuclei were defined with a blue DAPI staining and labeling of endothelial cells, macrophages and microglia was seen as a red fluorescence staining. APNG labeling in these non-tumor cells were automatically excluded in accordance with the analysis algorithm (Fig 2).

### APNG expression levels in WHO grade II tumors using qIHC

**Fig 3:**
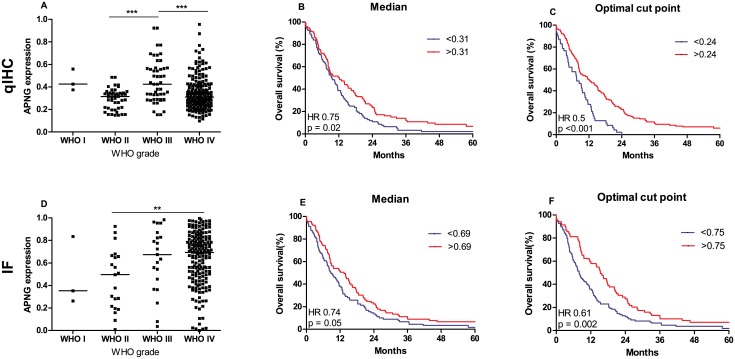
Box-plots and survival curves. Box-plots showing the level of APNG and survival curves for APNG qIHC (A+C+E) and IF (B+D+E). *qIHC*: There is no difference between the expression in level in patients with WHO grade IV tumors and patients with WHO grade I, II, and III (A). Divided at median APNG was prognostic (*P* = 0.02) (C). At optimal APNG cut point (25% vs 75% of the patients), HR improved (*P* < 0.001) (E). *IF*: The expression in patients with WHO grade IV tumors was higher than the expression in patients with low-grade gliomas, but similar to the expression in patients with WHO grade III tumors (B). Divided at median there was a trend towards APNG being prognostic (*P* = 0.05) (D). At optimal APNG cut point (61% vs. 39% of the patients), APNG was a prognostic factor in univariate analysis (*P* = 0.002) (F).

Median APNG expression level was 0.32 dots/cell (range 0.15–0.49) (Fig 3). No association between APNG expression and OS was identified (HR = 1.05; *P* = 0.93). Median OS was 62.4 months in patients with low expression of APNG and 57.2 months in patients with high expression. The prognostic significance of IDH1 was investigated. A total of 20 patients (80%) had mutated IDH1 (mIDH1). When patients were divided based on APNG and IDH1 status, no difference in OS was observed (*P* > 0.05).

### APNG expression levels in WHO grade III tumors using qIHC

Median APNG expression level was 0.42 dots/cell (range 0.15–0.92) (Fig 3). A trend towards better OS in patients with high APNG expression level was identified, median OS 21.4 months as compared to patients with low APNG expression, median OS 8.1 months (HR = 0.82; *P* = 0.67). When patients were divided based on APNG and IDH1 status, no difference in OS was observed (*P* > 0.05).

### APNG expression levels in WHO grade IV tumors using qIHC

Median APNG expression level was 0.31 dots/cell (range 0.099–0.96) (Fig 3) in the group of patients with WHO grade IV tumors. This was not significantly different from the expression in patients with WHO grade I, and II and III tumors (Fig 3). Using 0.31 as cutoff, a significant worse OS was observed in patients with low APNG expression level compared to patients with high expression (HR = 0.75; *P* = 0.02) (Fig 3). Median OS was 12.2 months in patients with high APNG and 9.4 months in patients with low APNG expression.

An explorative optimal cutoff analysis was performed. In qIHC 0.24 dots/cell was identified as the optimal cutoff, dividing patients into an APNG low (25% of the patients) and an APNG high group (75% of the patients). Patients with high expression of APNG had improved OS (HR = 0.50; *P* = < 0.001) (Fig 3). Similar results were seen in multivariate analysis adjusting for the effects of age, performance status, tumor crossing midline, treatment and gender (HR = 0.53; *P* = 0.001).

### APNG expression levels in WHO grade II tumors using IF

In the group of WHO grade II tumors, median AF was 0.50 (range 0.0091–0.93) (Fig 3). Patients with low AF had an improved survival; median OS 68.1 months compared to 56.3 in patients with high AF (HR = 1.91; *P* = 0.27). When patients were divided based on APNG and IDH1 status, no difference in OS was observed (*P* > 0.05).

### APNG expression levels in WHO grade III tumors using IF

Median AF was 0.67 (range 0.036–0.98) (Fig 3). Patients with high AF had a poor outcome (median OS 6.8 months) compared to patients with low AF (median OS 34.7 months) (HR = 2.46; *P* = 0.09). When patients were divided based on APNG and IDH1 status, no difference in OS was observed (*P* > 0.05).

### APNG expression levels in grade IV tumors using IF

Median AF of WHO grade IV tumors was 0.69 (range 0.0001–0.996) (Fig 3). This was higher than the expression in patients with low-grade gliomas, but similar to the expression in patients with WHO grade III tumors (Fig 3). Patients with high AF had the best outcome (HR = 0.74; *P* = 0.05) with a median OS of 13.4 months (Fig 3). Patients with low AF had a median OS of 8.7 months. In an explorative analysis dichotomizing at AF of 0.75 (61% of the patients with low AF and 39% with high AF) was identified as the optimal cutoff (HR = 0.61; *P* = 0.002) (Fig 3). When adjusting for the effect of clinical variables age, performance status, tumor crossing midline, treatment and gender a similar trend was observed (HR = 0.81; *P* = 0.24) (Table 2).

**Table 2:** Multivariate Cox regression analysis of APNG protein levels in glioblastomas including clinical parameters.

Variable	Baseline model			APNG qIHC			APNG IF		
	HR	95% CI	P-value	HR	95% CI	P-value	HR	95% CI	P-value
**Age**	1.01	1.00–1.03	0.23	1.01	1.00–1.03	0.15	1.01	1.00–1.03	0.07
**Gender**									
Female	1.00			1.00			1.00		
Male	1.53	1.11–2.10	0.009	1.45	1.05–2.00	0.02	1.36	0.96–1.95	0.09
**Performance status**									
0–1	1.00			1.00			1.00		
2–4	2.58	1.72–3.88	0.001	2.61	1.74–3.92	0.001	1.22	1.04–1.44	0.02
**Tumor crossing midline**									
No	1.00			1.00			1.00		
Yes	1.43	0.88–2.30	0.15	1.27	0.79–2.04	0.33	1.02	0.56–1.85	0.95
**Post-surgical treatment**									
Stupp	1.00			1.00			1.00		
Palliative	1.75	1.21–2.53	0.003	1.52	1.04–2.24	0.03	1.77	1.22–2.58	0.003
None	12.5	6.80–22.9	0.001	12.9	7.00–23.8	0.001	14.4	7.54–27.6	0.001
**APNG**									
Low	-	-	-	1.00			1.00		
High				0.53	0.36–0.77	0.001	0.81	0.57–1.15	0.24

Abbreviations: qIHC quantitative immunohistochemistry; IF immunofluorescence. APNG low/high according to optimal cut point.

### MGMT status and survival

**Fig 4:**
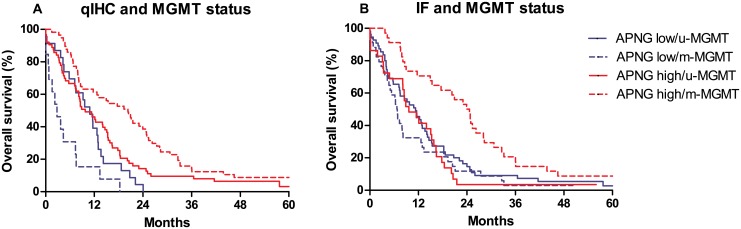
Survival curves based on APNG and MGMT status. Median survival in patients with low APNG and methylated MGMT promoter was 2.8/6.1 months (qIHC/IF) compared to 4.5/11.3 months in patients with low APNG expression and a un-methylated MGMT promoter. Patients with high APNG expression and un-methylated MGMT promoter had a median overall survival of 9.8/9.6 months whereas patients with high APNG expression and methylated MGMT promoter (H/M) had a median overall survival of 20.3/20.3 months (*P* < 0.001). In multivariate analysis patients with high APNG expression and methylated MGMT promoter demonstrated a better overall survival than patients with low APNG expression and methylated MGMT promoter; HR 0.55, *P* = 0.04 (qIHC) and HR = 0.66, *P* = 0.1 (IF). Abbreviations: u-MGMT un-methylated MGMT promoter, m-MGMT methylated MGMT promoter. APNG low/high was defined according to the optimal cut point, which was for qIHC was 0.24 and for IF was 0.75.

When GBM patients were divided based on APNG (optimal cutoff) and MGMT status, a significant difference in OS was identified; patients with high APNG expression and methylated MGMT promoter had a median OS of 19.8 months, and patients with low APNG expression and a methylated MGMT promoter had a median OS of 11.2 months (*P* < 0.001). This was also significant in multivariate analysis, where patients with high APNG expression and a methylated MGMT promoter demonstrated a better OS, than patients with low APNG expression and a methylated MGMT promoter (HR = 0.59; *P* = 0.08). However, APNG expression was not prognostic in patients with an un-methylated MGMT promoter (HR = 0.80; *P* = 0.37) (Fig 4).

Using IF similar results were obtained; median OS was 24.3 months in patients with high APNG expression and methylated MGMT status and 11.3 months in patients with low APNG and a non-methylated MGMT promoter (*P* < 0.001). A similar trend was found in multivariate analyses (HR = 0.64; *P* = 0.08). In patients with an un-methylated MGMT promotor APNG was not prognostic (HR = 1.21; *P* = 0.42) (Fig 4).

### APNG expression in the TCGA-database

**Fig 5:**
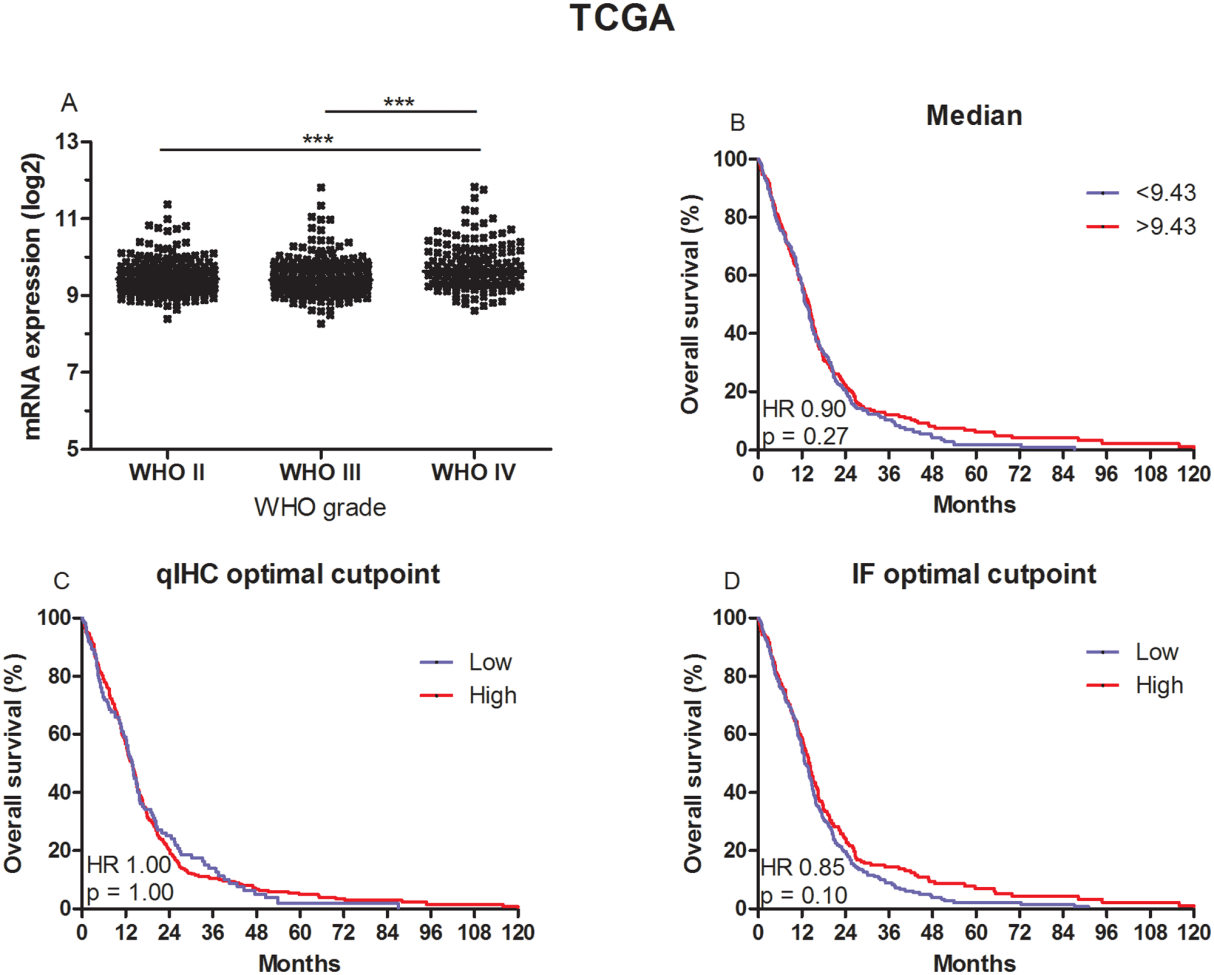
Box-plot and survival curves for patients in the TCGA data set. The level of APNG mRNA was significantly higher in patients with WHO grade IV tumors compared to patients with WHO grade II and III tumors (A). When patients were divided at the median (B) or at the optimal cut point for qIHC (25% of the patients with the lowest APNG vs. the 75% of the patients with the highest APNG level) (C), APNG mRNA was not associated with overall survival. When divided at the optimal cut point for IF (61% of the patients with low APNG vs. the 39% with high APNG), a trend towards high APNG and improved overall survival (HR = 0.85; *P* = 0.10) was identified (D).

In the WHO grade II-IV cohort the median levels of APNG mRNA were 9.43 (range 8.39–11.38), 9.41 (range 8.27–11.81), and 9.63 (range 8.61–11.83) for patients with WHO grade II, III and IV tumors (Fig 5). The level of APNG mRNA was significantly higher in patients with WHO grade IV tumors compared to patients with WHO grade II and III tumors (*P* < 0.001).

In the GBM cohort the median level of APNG mRNA was 7.32 (6.10–9.07). The level of APNG mRNA was not associated with OS when divided at the median (HR = 0.90; *P* = 0.27) or at the optimal cutoff for qIHC (25% of the patients with the lowest APNG vs. the 75% of the patients with the highest APNG level) (HR = 1.00; *P* = 1.00). However; when divided at the optimal cutoff for IF (61% of the patients with low APNG vs. the 39% with high APNG), a trend towards high APNG and improved OS (HR = 0.85; *P* = 0.10) was identified (Fig 5). For all 3 cut points we performed a multivariate analysis including age, gender and MGMT status, which indicated a better survival in patients with high levels of APNG mRNA, but this was not significant (S3 Table).

## Discussion

This is the first study to investigate the prognostic value of APNG with both qIHC and quantitative IF in GBMs with simultaneous exclusion of APNG expression in non-tumor cells. We demonstrated that GBM patients with high APNG expression had an improved OS compared to patients with low expression. This was shown in univariate analysis and was confirmed in multivariate analysis in the qIHC assay. Statistical significance was, however, only observed in univariate and not in multivariate analysis using IF. When dichotomizing patients into high- and low expressers with an optimal cutoff, a significant association between OS and APNG was found in both assays. No statistically significant association between APNG-expression and OS in patients with WHO grade II and III tumors was observed, probably due to the small number of patients. Surprisingly: in patients with WHO grade III tumors the IF assay revealed a trend towards improved OS in patients with low APNG expression. This is in contrast to the other results. However, the analysis included only few patients, which may influence the results.

Using qIHC, APNG expression was higher for WHO grade III tumors than for both WHO grade II and grade IV tumors. This was in contrast to our results obtained using IF as well as TCGA data and the study by Liu et al [18]. The higher levels in grade III tumors observed using qIHC may be caused by the high levels observed in the few anaplastic oligodendrogliomas. Some of these patients were excluded from the IF analysis due to technical errors. As the number of patients in our analysis was small, exclusion of even few patients, especially patients with very high levels of APNG, may influence the results significantly. This may explain the conflicting results in patients with WHO grade III tumors, where nearly 20% of the patients in the qIHC analyses were excluded from the IF approach. Moreover, differences in the sensitivity of the two assays used in our study may influence the results. Accordingly, the sensitivity of the IF approach may be higher in terms of detecting high grade tumors with high APNG protein levels. This needs validation in a larger cohort.

Using both qIHC and IF, tumors with the same WHO grade had large differences in APNG expression levels. This is in accordance with observations made by others, where e.g. APNG mRNA levels are reported to vary up to tenfold between GBMs [29]. This is not only true for APNG. In the clinical setting MGMT is often categorized as positive or negative, however, MGMT has been reported to exhibit varying levels of expression within the same WHO grade [30–32]. The different expression levels illustrates the highly heterogeneous nature of gliomas, GBMs especially, and support the need for more individualized approaches [33].

For patients with WHO grades II and III tumors, differences in OS were observed when dichotomizing at the median expression, favoring patients with high expression, except for patients with WHO grade III tumors when using IF. This trend towards improved survival is supported by the results for WHO grade IV. The relatively small patient groups of lower grade tumors might contribute to the lack of statistical significance, and a larger patient group is required to further evaluate the prognostic value of APNG in WHO grade II and III tumors. As IDH1 has been shown to be prognostic in patients with WHO grade II and III tumors, we investigated if the combined information of APNG and IDH1 in these patients was prognostic. This was not confirmed, probably due to the limited number of patients in each group.

In the largest patient group of WHO grade IV tumors, we found a significant association between high levels of APNG and better OS when adjusting for clinical parameters as well as MGMT status. All though based on mRNA, the results were supported by the results from the TCGA dataset which indicate a similar trend in a multivariate analysis when using the cutoff from the IF assay. Moreover our results are in line with results obtained in *in vitro* studies [29, 34], where high APNG levels were associated with increased sensitivity to treatment. However, this is in contrast to the study of Liu et al. showing that patients with no APNG expression had the most favorable outcome [18]. Results from that study should, however, be interpreted with caution since all glioma grades were combined into one analysis, and as it was demonstrated in our study, high grade gliomas expressed higher APNG levels than low grade gliomas. Our study is the first to exclude APNG expression in non-tumor cells from the analysis. The rationale for doing so is “false positive” staining in non-neoplastic cells influencing the measurements. This may also contribute to the discrepancy between our study and the study by Liu et al. In the study by Agnihotri et al., 244 GBMs were separated as being APNG-negative and APNG-positive using conventional IHC. However, different results were obtained in their study as high expression of APNG was associated with poor OS in some of the cohorts while there was no difference in OS in other cohorts [3]. Moreover, no multivariate analyses were reported. Using the categories “positive” and “negative” only, instead of continuous quantitative measurements may be an important explanation for the different results obtained, as the cutoffs might differ between the studies.

In our study no tumors were APNG-negative. This might be due to the high sensitivity of both of our methods and suggests a need for differentiation between expression levels. Another parameter, which may influence the results obtained in different studies, is the amount of tissue analyzed for each tumor. We used whole slides and observed large intra-tumoral variation in APNG expression. Therefore we excluded patients with small amounts of tissue. In the study by Agnihotri et al., tissue micro-arrays were used, and the individual cores were categorized as “positive” and “negative” [3]. Previous studies have shown that tissue micro-arrays should be used with caution [35, 36], and the use of tissue micro-arrays could possibly explain the difference between our results and the results obtained by Agnihotri et al. Discrepancy between the different studies may also be influenced by different antibody clones used. Several different clones have been used in APNG studies, and only Liu et al. used the same clone as the one used in our study (Abcam clone ab55461). To minimize the degree of uncertainty associated with different clones further studies are needed.

Based on the supposed function of APNG as a part of the BER system, it seems probable that a lack of this enzyme would confer poorer resistance towards alkylating agents inducing N3- and N7 lesions to the DNA. A previous study has shown that destabilization of the BER system confers increased sensitivity towards alkylating agents in human breast cancer cells [12]. The BER system appears to be sensitive to high amounts of its critical enzymes. Thus, overexpression of molecules in one part of the system might influence the subsequent steps, which no longer can cope with the increased amount of substrate. High expression of APNG may cause this destabilization, which is explained by a downstream shift of the rate limiting step of the system. High numbers of DNA alkylations being handled by a high level of APNG accordingly puts pressure on the downstream enzymes of the BER system. The critical enzyme, Polymerase β, can become over-saturated and give rise to an increased amount of cytotoxic 5’dRP intermediates [12, 13]. It has been suggested that these cytotoxic lesions, which may be more toxic than the original lesions, lead to apoptosis of tumor cells, thereby resulting in an improved patient OS [15]. Our protein data and to some extent the TCGA data are in line with these aspects of the APNG biology, as high APNG protein and mRNA levels were associated with improved OS. In a related study on ovarian cancer cell lines, the authors found that cells expressing high levels of APNG had an increased sensitivity towards TMZ. This suggests that the BER system is a critical system in terms of survival of tumor cells exposed to alkylating agents [11]. It may be speculated if APNG expression does not influence survival when MGMT is expressed in GBM patients with unmethylated promoters, and that the O6-adducts repaired by MGMT are more cytotoxic than the N-alkylations repaired by APNG [37]. This may explain why our results indicate that the significance of the BER system appear higher in MGMT promoter-methylated GBMs.

In conclusion APNG measured by qIHC was found to be a significant independent prognostic factor for OS in patients with GBMs. This was supported by IF measurements. The quantitative approach combined with exclusion of APNG expression in non-tumor cells in both assays have contributed to a more exact analysis of the tumor-specific expression. We expect that APNG qIHC can potentially identify GBM patients who will not benefit from treatment with TMZ.

## Supporting information

S1 Table Patient characteristics in the TCGA dataset. Patient characteristics for the 620 patients evaluated by RNA sequencing analyses.
(DOCX)

S2 Table Patient characteristics in the TCGA GBM dataset. Patient characteristics for the 497 GBM patients in whom APNG status was generated using Affymetrix.
(DOCX)

S3 Table Multivariate analyses based on the TCGA GBM dataset. (DOCX)
