# Supplementary material for: APNG as a prognostic marker in patients with glioblastoma
Source: PLoS One. 2017 Jun 29;12(6):e0178693. doi: 10.1371/journal.pone.0178693 (PMC5490991; doi:10.1371/journal.pone.0178693)
Supplement: S1 Table — Patient characteristics for the 620 patients evaluated by RNA sequencing analyses. (DOCX) [file pone.0178693.s001.docx]

|  | WHO Grade II | |  | WHO Grade III | |  | WHO Grade IV | |
| --- | --- | --- | --- | --- | --- | --- | --- | --- |
|  | n | % |  | n | % |  | n | % |
| **Subjects** | 226 |  |  | 244 |  |  | 150 |  |
| **Age** (median) | 38 |  |  | 44 |  |  | 60 |  |
| **Gender** |  |  |  |  |  |  |  |  |
| Male | 117 | 52 |  | 137 | 56 |  | 97 | 65 |
| Female | 96 | 42 |  | 102 | 42 |  | 52 | 35 |
| Unknown | 13 | 6 |  | 5 | 2 |  | 1 | 1 |
| **IDH status** |  |  |  |  |  |  |  |  |
| Mutated | 206 | 91 |  | 178 | 73 |  | 10 | 7 |
| Wildtype | 18 | 8 |  | 66 | 27 |  | 137 | 91 |
| Unknown | 2 | 1 |  | 0 |  |  | 3 | 2 |
| **mRNA APNG** (median) | | | |  |  |  |  |  |
|  | 9.43 |  |  | 9.41 |  |  | 9.63 |  |
| **Dead** | 32 | 14 |  | 71 | 29 |  | 117 | 78 |
| **OS** (Months) | 24 |  |  | 19 |  |  | 11 |  |
