# Supplementary material for: APNG as a prognostic marker in patients with glioblastoma
Source: PLoS One. 2017 Jun 29;12(6):e0178693. doi: 10.1371/journal.pone.0178693 (PMC5490991; doi:10.1371/journal.pone.0178693)
Supplement: S2 Table — Patient characteristics for the 497 GBM patients in whom APNG status was generated using Affymetrix. (DOCX) [file pone.0178693.s002.docx]

|  | WHO Grade IV | |  |
| --- | --- | --- | --- |
|  | n | % |  |
| **Subjects** | 497 |  |  |
| **Age** (median) | 60 |  |  |
| **Gender** |  |  |  |
| Male | 307 | 62 |  |
| Female | 189 | 38 |  |
| Unknown | 1 | <1 |  |
| **MGMT status** | |  |  |
| Methylated | 158 | 32 |  |
| Unmethylated | 168 | 34 |  |
| Unknown | 171 | 33 |  |
| **mRNA APNG** (median) | | | |
|  | 7.32 |  |  |
| **Dead** | 420 | 85 |  |
| **OS** (Months) | 12 |  |  |
