# Supplementary material for: APNG as a prognostic marker in patients with glioblastoma
Source: PLoS One. 2017 Jun 29;12(6):e0178693. doi: 10.1371/journal.pone.0178693 (PMC5490991; doi:10.1371/journal.pone.0178693)
Supplement: S3 Table — (DOCX) [file pone.0178693.s003.docx]

|  | Baseline (n=325) | |  | Median (n=325) | | | |  | qIHC (n=325) | |  | IF (n=325) | |
| --- | --- | --- | --- | --- | --- | --- | --- | --- | --- | --- | --- | --- | --- |
|  | HR | P-value |  | HR | | P-value | |  | HR | P-value |  | HR | P-value |
| **Age** | 1.03 | <0.001 |  | 1.03 | | <0.001 | |  | 1.03 | <0.001 |  | 1.03 | <0.001 |
| **Gender** | | |  | |  | |  | | | | | | |
| Male | 1.00 |  |  | 1.00 | |  | |  | 1.00 |  |  | 1.00 |  |
| Female | 0.66 | 0.003 |  | 0.66 | | 0.003 | |  | 0.67 | 0.003 |  | 0.66 | 0.002 |
| **MGMT** | | | | | | | | | | | | | |
| Unmethylated | 1.00 |  |  | 1.00 | |  | |  | 1.00 |  |  | 1.00 |  |
| Methylated | 0.77 | 0.05 |  | 0.77 | | 0.04 | |  | 0.76 | 0.04 |  | 0.77 | 0.04 |
| **APNG** | | | | | | | | | | | | | |
| Low | - | - |  | 1.00 | |  | |  | 1.00 |  |  | 1.00 |  |
| High | - | - |  | 0.94 | | 0.64 | |  | 0.84 | 0.21 |  | 0.83 | 0.17 |
